# Risk Evaluation of Pollutants Emission from Coal and Coal Waste Combustion Plants and Environmental Impact of Fly Ash Landfilling

**DOI:** 10.3390/toxics11040396

**Published:** 2023-04-21

**Authors:** Jovana Z. Buha Marković, Ana D. Marinković, Jasmina Z. Savić, Milica R. Mladenović, Milić D. Erić, Zoran J. Marković, Mirjana Đ. Ristić

**Affiliations:** 1 Vinča Institute of Nuclear Sciences—National Institute of the Republic of Serbia, University of Belgrade, Mike Petrovića Alasa 12–14, 11351 Belgrade, Serbia; aradojevic@vin.bg.ac.rs (A.D.M.); jasnas@vin.bg.ac.rs (J.Z.S.); mica@vin.bg.ac.rs (M.R.M.); milic@vin.bg.ac.rs (M.D.E.); zoda_mark@vin.bg.ac.rs (Z.J.M.); 2 Faculty of Technology and Metallurgy, University of Belgrade, Karnegijeva 4, 11120 Belgrade, Serbia; risticm@tmf.bg.ac.rs

**Keywords:** coal and coal waste combustion, emission, gaseous pollutants, particulate matter, trace elements, PAHs, environmental risk estimation, fly ash disposal, lead isotopic fingerprint

## Abstract

Emission factors (EFs) of gaseous pollutants, particulate matter, certain harmful trace elements, and polycyclic aromatic hydrocarbons (PAHs) from three thermal power plants (TPPs) and semi-industrial fluidized bed boiler (FBB) were compared. EFs of particulate matter, trace elements (except Cd and Pb), benzo[a]pyrene, and benzo[b]fluoranthene exceed the upper limits specified in the EMEP inventory guidebook for all combustion facilities. The comparison of trace elements and PAHs content in fly ashes (FAs) from lignite and coal waste combustion in TPPs and FBB, respectively, as well as the potential environmental impact of FAs disposal, was performed by employing a set of ecological indicators such as crustal enrichment factor, risk assessment code, risk indices for trace elements, and benzo[a]pyrene equivalent concentration for PAHs. Sequential analysis shows that the trace elements portion is the lowest for water-soluble and exchangeable fractions. The highest enrichment levels in FAs are noticed for As and Hg. Based on toxic trace elements content, FAs from TPPs represent a very high ecological risk, whereas fly ash from FBB poses a moderate ecological risk but has the highest benzo[a]pyrene equivalent concentration, indicating its increased carcinogenic potential. Lead isotope ratios for Serbian coals and FAs can contribute to a lead pollution global database.

## 1. Introduction

Coal combustion is both the primary energy source in many countries and one of the major anthropogenic sources of atmospheric, water, and soil pollution. It induces a variety of environmental and health issues due to the release of gaseous pollutants such as sulfur dioxide (SO_2_), nitrogen oxide (NOx), carbon monoxide (CO), and solid particulate matter (PM) [1]. Millions of tons of coal are burned in different power plants worldwide [2], resulting in enormous quantities of combustion residues, such as fly and bottom ashes. Branch TPP Nikola Tesla (TPPs Nikola Tesla A and B, Kolubara A and Morava) generates more than 50% of electricity, while TPPs Kostolac A and B produce around 17% of overall electricity in Serbia [3]. Approximately 40 million tons of lignite from the Kolubara and Drmno basins are burned in thermal power plants, producing 6 million tons of FAs [4]. Since coal resources are limited [5], using alternative fuels such as coal waste in various combustion technologies is increasingly important. Fluidized bed combustion has proven to be an efficient and environmentally acceptable technique for producing energy from low quality coals because it operates at a lower temperature (850°C) and has the possibility of efficient in-bed desulphurization, resulting in lower NOx and SO_2_ emissions [6,7]. 

Coal combustion is a significant source of gaseous pollutants (SO_2_, NOx, CO), PM, as well as trace elements and organic pollutants emissions [8]. Emission measurements of SO_2_, NOx, CO, and PM are needed due to their impact on air pollution and given emission limits [9]. The trace elements are categorized into three groups based on their partitioning behavior during coal combustion [10,11]: extremely volatile (such as Hg), prone to condense on fly ash particles (As, Pb, Cd), and uniformly distributed among BAs and FAs (Cu, Ni and Cr). In addition, some portions of these element compounds are discharged into the atmosphere along with flue gases. Therefore, their monitoring is significant since these fine particles can travel long distances. Although the partitioning of PAHs in flue gasses along with emitted particles has been studied [12,13], investigations on the PAHs emission characteristics from different boilers and combustion technologies are still scarce. 

Ash content depends on coal type and combustion conditions, such as temperatures, air flow, type of combustion, etc. [14,15]. Many potentially hazardous substances, such as certain trace element compounds and/or semi-volatile and volatile organic compounds, can be concentrated in bottom ash (BA), FA, and fine particles, posing a significant threat to the environment [16,17]. The factors that have the greatest influence on the content of these compounds include their initial content in feed fuel, combustion conditions, and the effectiveness of the particulate control device used [18,19,20]. 

Sequential extraction is a method for assessing the chemical form, mobility, and physicochemical and biological availability of trace elements [21,22]. Different tools such as the crustal enrichment factor (CEF), the risk assessment code (RAC), and the risk index (RI) can be applied to calculate the specific element enrichment, prospective leachability of a particular pollutant, and overall ecological risk [23,24,25]. 

PAHs belong to persistent hazardous organic compounds, and the United States Environmental Protection Agency classifies 16 of them as priority pollutants [26]. Additionally, benzo[a]pyrene-based toxic equivalent factor (BaP_eq_) and benzo[a]pyrene-based equivalent carcinogenic power (BaPE) are widely used to assess the potential harm of PAHs to the environment [27,28]. These values enable the estimation of the overall environmental risk of investigated fly ashes.

Among many toxic elements, lead is a major global pollutants. Natural lead is normally composed of four stable isotopes with masses of 204, 206, 207, and 208 [29]. To trace back and identify the source of lead pollution, such as mining, industry, and coal combustion [30,31], it is essential to know the lead fingerprint (the isotope ratios). The isotopic composition of Pb is usually expressed by the following ratios ^206^Pb/^204^Pb, ^206^Pb/^207^Pb, ^208^Pb/^206^Pb, ^208^Pb/^207^Pb, with the ^206^Pb/^207^Pb being the most commonly used. There are studies on lead isotope ratios in coals and other pollution sources [30,32,33], but there is a lack of information concerning lead isotopes in Serbian coals and FAs. 

In this study, the overall environmental impact from electricity production in TPPs Kolubara A, Kostolac B, Nikola Tesla A, and semi-industrial FBB, as combustion facilities with different combustion regimes, fuel types, and capacities were compared. The purpose of this research was to determine the emission of SO_2_, NOx, CO, and total PM to estimate emission factors of the most harmful trace elements and PAHs, as well as to evaluate their values with the EMEP/EEA permissible limits. Furthermore, the goal was to assess the potential environmental risk posed by the most harmful substances if ash is disposed on landfills and to update a broad database on lead isotope ratios for Serbian coals and FAs.

## 2. Materials and Methods

### 2.1. Ash Sample Collection and Storage

In this study, bulk fly ashes from TPPs Kolubara A (TPPKb), Kostolac B (TPPKs), Nikola Tesla A (TPPNT), and from the cyclone of fluidized bed boiler (CFB) were collected and examined. For lead isotopic measurements, representative coal samples (4), as well as FAs from TPPs and FBB (8 samples) were analyzed. Sampling and sample preparation followed a previously described methodology [34]. Table S1 in Section S1 (Supplementary) contains the details about sampling locations.

### 2.2. Emission of NOx, CO, SO_2_ and Total PM from TPPs and FBB

The content of flue gas pollutants was determined according to the standards [35,36,37,38,39,40]. The flue gases sampling was carried out at the stack or at the flue gas line in front of the stack. Multicomponent gas analyzer Horiba PG 350E (O_2_, CO_2_, CO, NOx, and SO_2_), gas conditioning unit PSS5 M&C Tech group and heated hose JCT Analysentechnik GmbH were used. Utilized gas sampling equipment was in accordance with standards [35,36,37,39,40]. Flue gas flows for all TPPs and FBB are shown in Table 1. 

Particulate matter sampling was performed by the isokinetic sampling system Heated Paul Gothe gas sample probe with a fine filter and pre-filter [38]. Total PM included all particulate matter with diameters higher than 0.3 µm. Sampling flow range: 0.5–4.0 m^3^/h.

### 2.3. Determination of Trace Elements and Lead Isotopic Ratios 

#### 2.3.1. Sequential Extraction of FAs 

The sequential extraction of trace elements (As, Be, Cd, Co, Cr, Cs, Cu, Ga, Ge, Hg, Mn, Mo, Ni, Pb, Sb, Sr, U, and V) was performed as described in the literature [41] with a modified last step. Details are explained in Section S2 (Supplementary).

#### 2.3.2. ICP-MS Analysis

Trace elements concentrations were determined by the inductively coupled plasma mass spectrometry (ICP-MS) using Agilent 7500ce instrument equipped with Octopole Reaction System in FullQuant mode. ICP-MS calibration was performed by Agilent Multi-Element Calibration Standards. Standard solutions and blanks were prepared in 2% HNO_3_. The analyses of each fraction (F1–F6) were performed in 3 replicates. The isotope analysis mode was used for lead isotopic measurements. The accuracy of the isotope ratio measurements was evaluated by analyzing a certified isotopic standard NIST SRM 981. External corrections due to a mass bias of the ICP-MS were performed by measuring a 5 g/L solution of NIST SRM 981. Mass bias correction factors were automatically calculated for the ratios ^207^Pb/^206^Pb (0.9925) and ^208^Pb/^207^Pb (0.9968). Other details about the ICP-MS analysis are described in Section S3 (Supplementary). 

### 2.4. Analysis of PAHs Content in FAs 

#### 2.4.1. PAHs Extraction

The extraction of PAHs from FAs was performed by a solid–liquid extraction. More details about PAHs extraction are provided in [34].

#### 2.4.2. HPLC Analysis

A Thermo Fisher Scientific Dionex UltiMate 3000 HPLC system with a diode array detector was applied to determine 16 priority PAHs along with two naphthalene substituted derivatives (1-methyl naphthalene and 2-methyl naphthalene). The details of the modified HPLC analysis are provided in [34].

### 2.5. Data Analysis

#### 2.5.1. Estimation of EFs for Selected Trace Elements and PAHs

Literature data [10,11,18,42,43,44] and data acquired from the field measurements (flue gas velocity, cross-sectional area, coal consumption, and ash production) were employed to estimate As, Cd, Cr, Cu, Hg, Ni, and Pb emission factors, as well as EFs for benzo[b]fluoranthene (BbF), benzo[k]fluoranthene (BkF), benzo[a]pyrene (BaP), and indeno [1,2,3-cd]pyrene (IP). The calculated EFs were compared with representative values provided in the EMEP/EEA guidebook [45].

#### 2.5.2. Crustal Enrichment Factor (CEF)

CEF specifies the degree to which each trace element in fly ash is enriched relative to its content crustal core. To reduce the alterations among diverse samples, CEF values are estimated in relation to a reference element with low occurrence variability. In this paper, *Mn* is used as a reference element. *CEF_n/Mn_* is calculated using the following formula:

(1) CEFn/Mn=CnCMnfly ashCnCMncrustal core

where *C_n_* is the concentration of each investigated trace element and *C_Mn_* is the concentration of manganese, both in *fly ash* samples and *crustal core*, respectively.

#### 2.5.3. Risk Assessment Code (RAC)

Usually, RAC defines the potential environmental risk of trace elements leaching from complex matrices, such as ashes [24,46]. It is calculated as the ratio of the sum of the water-soluble fraction (F1), the exchangeable fraction (F2), and the carbonate bound fraction (F3) to the total element concentration in a representative sample, expressed in percentage.

#### 2.5.4. Pollution Index (PI)

PI identifies the pollution level of all investigated trace elements that can pose harm to the soil [47]. PI is calculated by the following equation:

(2) $$PI^{i}=\frac{C_{n}}{C_{b}}$$

where *PI^i^* is the single pollution index; and *C_n_* and *C_b_* are the elemental concentration in fly ash and crustal core, respectively.

#### 2.5.5. Risk Index (RI)

RI estimates the individual ecological risk level for the most hazardous trace elements (As, Cd, Co, Cr, Cu, Hg, Ni, and Pb), while *RI_sum_* evaluates the overall ecological risk of the investigated sample. It is calculated by the following equations:

(3) $$RI^{i}=T_{r\;}^{i}\times PI^{i}$$

(4) $$RI_{sum}=\overset{n}{\underset{i=1}{\sum }}RI^{i}$$

where *T_r_* represents the toxic response for each potentially toxic trace element [23].

#### 2.5.6. *BaP_eq_* and BaPE Values for FAs

*BaP_eq_* is used to estimate the PAHs overall toxic potency in investigated samples. *BaP_eq_* is determined according to the following equation:

(5) $$BaP_{eq}=\overset{18}{\underset{i=1}{\sum }}TEF_{i}\times c_{i}$$

where *c_i_* is the individual PAH concentration, and *TEF_i_* represents the toxic equivalency factor of each PAH [48].

BaPE is calculated according to the following equation [20]:

(6) BaPE =BaA×0.06+BbF ×0.07+BkF ×0.06+BaP+DahA ×0.6++ IP ×0.08

## 3. Results and Discussion

### 3.1. NOx, CO, SO_2_ and Total PM Emission from TPPs and FBB

**Figure 1:**
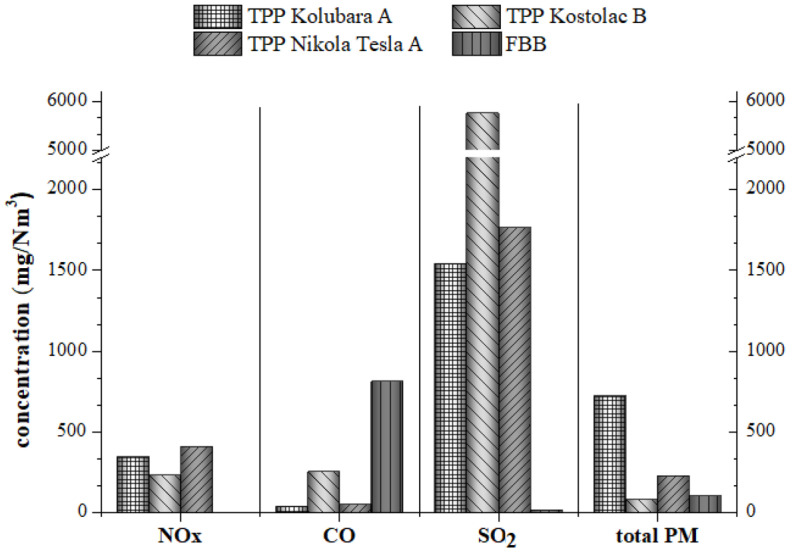
NOx, CO, SO_2_, and total PM concentrations (all in mg/Nm^3^) in flue gases from TPP Kolubara A, TPP Kostolac B, TPP Nikola Tesla A, and FBB.

The highest NOx concentration was noticed for TPP Nikola Tesla A, while total PM concentration was the highest for TPP Kolubara A (Figure 1). The increased content of SO_2_ in TPP Kostolac B surpasses its corresponding limit value (listed in Table S2) [49]. Higher sulfur content in lignite utilized in TPP Kostolac B most likely leads to elevated SO_2_ content in the flue gas. CO is higher in FBB than in other combustion facilities, possibly due to incomplete combustion and lower combustion temperature in FBB (Figure 1). The electrostatic precipitators installed in TPPs have high efficiency (more than 99%) in collecting particulate matter, but they are usually inefficient for capturing fine and/or ultrafine particles [50,51].

The EF values of all investigated pollutants are listed in Table 2, along with their corresponding lower and upper limits and the average value obtained from the EMEP inventory guidebook. EFs for total particulate matter (Table 2A) in flue gases for all combustion facilities exceed their representative EMEP PM10 upper limits fivefold (for TPP Kolubara A). The highest values of EFs for other pollutants were observed for CO in TPPs Nikola Tesla A and Kostolac B, as well as for FBB and NOx in TPP Kolubara A, and SOx in TPPs Kostolac B and Kolubara A.

### 3.2. Sequential Extraction of Trace Elements from FAs

**Figure 2:**
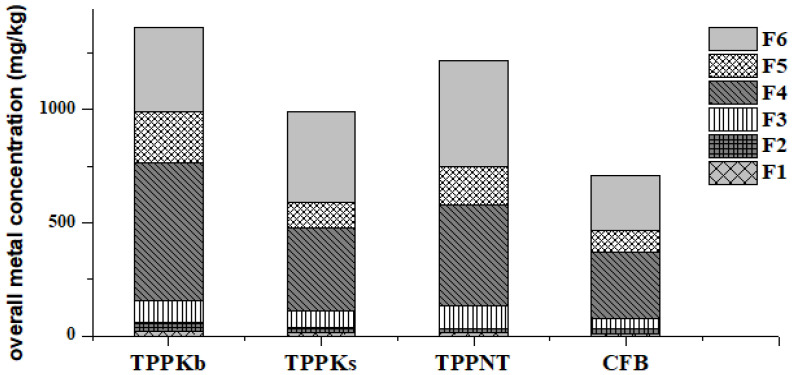
Overall trace element concentrations in F1-F6 fractions of fly ashes (TPPKb, TPPKs, TPPNT, and CFB).

The chemical speciation of 18 trace elements in FAs between the water-soluble fraction (F1), the exchangeable fraction (F2), the carbonate bound fraction (F3), the metal oxide bound fraction (F4), the organic bound fraction (F5), and the residual fraction (F6) was performed. Overall trace element concentrations within six fractions (Figure 2) range from 709.81 mg/kg in CFB to 1360.90 mg/kg in TPPKb. The lowest concentrations are within F1 fractions for all FAs, going from 9.35 mg/kg in CFB to 22.45 mg/kg in TPPKb. The highest concentrations are noticed for F4 fractions in TPPKb and CFB and F6 fractions in TPPKs and TPPNT.

**Figure 3:**
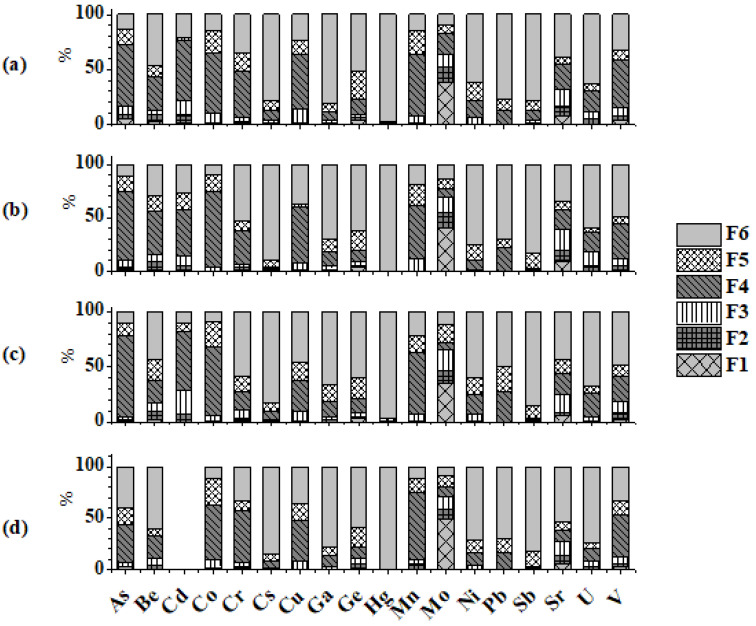
Distribution of investigated trace elements in fly ashes: TPPKb (**a**), TPPKs (**b**), TPPNT (**c**), and CFB (**d**) among the water-soluble fraction (F1), the exchangeable fraction (F2), the carbonate bound fraction (F3), the metal oxide bound fraction (F4), the organic bound fraction (F5), and the residual fraction (F6).

The distribution of all investigated elements among fractions (F1–F6) is shown in Figure 3. The water-soluble fraction is of major environmental concern since anions, such as chlorides and sulfates, are easily available for plant and soil uptake [54]. According to Figure 3, most compounds of the studied trace elements are not water-soluble, so portions of their F1 fractions are low. The exception is molybdenum, which can easily form water-soluble species [15].

In addition, some of the investigated elements (Figure 3) in all FAs (Cs, Ga, Ge, Hg, Ni, Sb, and U) have low levels in F1 and F2 fractions as shown in the literature [46]. Among previously mentioned elements, germanium prevails in the water-soluble phase up to 3.33% in TPPKs, probably due to the presence of GeO_2_ and GeS_2_ [55]. In F2 fractions, the proportions of Mo and Sr are the highest (from 3.0% for Sr in TPPNT to 15.42% for Mo in TPPKs). Sr solubility is likely due to its cationic leaching pattern and removal of the exchangeable cation sorbed on the ash particles surface [56]. F3 fraction mainly consists of elements as carbonates or oxides/hydroxides (Cd, Cu, Mn, Mo, V, and Sr) [57,58]. pH reduction increases the mobility of the carbonate form of these trace elements [59]. Trace elements in the F4 extraction step are moderately mobile and sensitive to redox potential changes [21]. The investigated elements proportion in F4 fractions ranges from 0.74% for Hg (TPPKb) to 73.62% for As (TPPNT). Elements of the F5 fraction can occur as oxidizable minerals, e.g., sulfides [22], and their mobility is relatively low unless they undergo the oxidation process. Co has a maximal portion of 26.45% in CFB among all elements within F5 fractions. The majority of elements have the highest distributions in residual fractions in all FAs (Cs, Ga, Ge, Hg, Ni, Pb, Sb, Sr, and U) or are evenly distributed between F4 and F6 fractions (As, Cr, Cu, and V). Since elements from the residual fractions have strong bonds with the mineral crystal lattice, their mobility is low, and they are not considered to be of environmental concerns.

**Figure 4:**
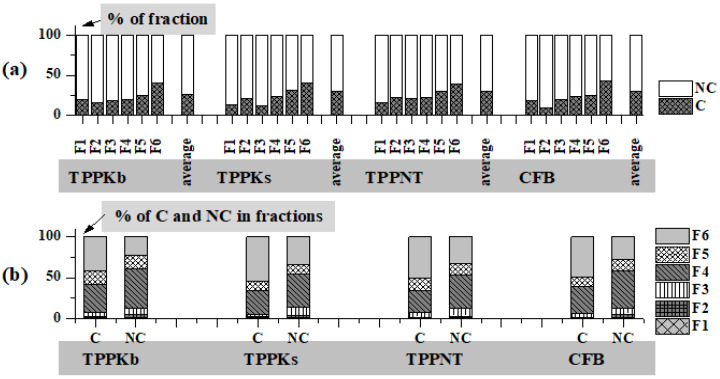
(**a**) Portions for carcinogenic (C) and non-carcinogenic elements (NC) and average values in each fraction for all fly ashes (TPPKb, TPPKs, TPPNT, and CFB); (**b**) overall distribution of C and NC elements among six fractions.

Among investigated elements, As, Cd, Cr, Hg, Ni, and Pb are carcinogenic (C), while Be, Co, Cs, Cu, Ga, Ge, Mn, Mo, Sb, Sr, U, and V are considered to be non-carcinogenic (NC). The carcinogenic element prevails in F6 fractions and goes from 39.52% in TPPNT to 42.92% in CFB, while the non-carcinogenic portion is up to 91.31% in the F2 fraction of CFB (Figure 4a). Figure 4b displays the distributions of the C and NC elements among the six fractions with the overall content set to be 100%. Carcinogenic element portions in the environmentally significant fractions (F1–F3) range from 5.07% in TPPKs to 7.76% in TPPKb, while non-carcinogenic element distributions vary from 12.23% in TPPNT to 13.50% in TPPKs (Figure 4b).

### 3.3. Lead Isotope Ratios

**Figure 5:**
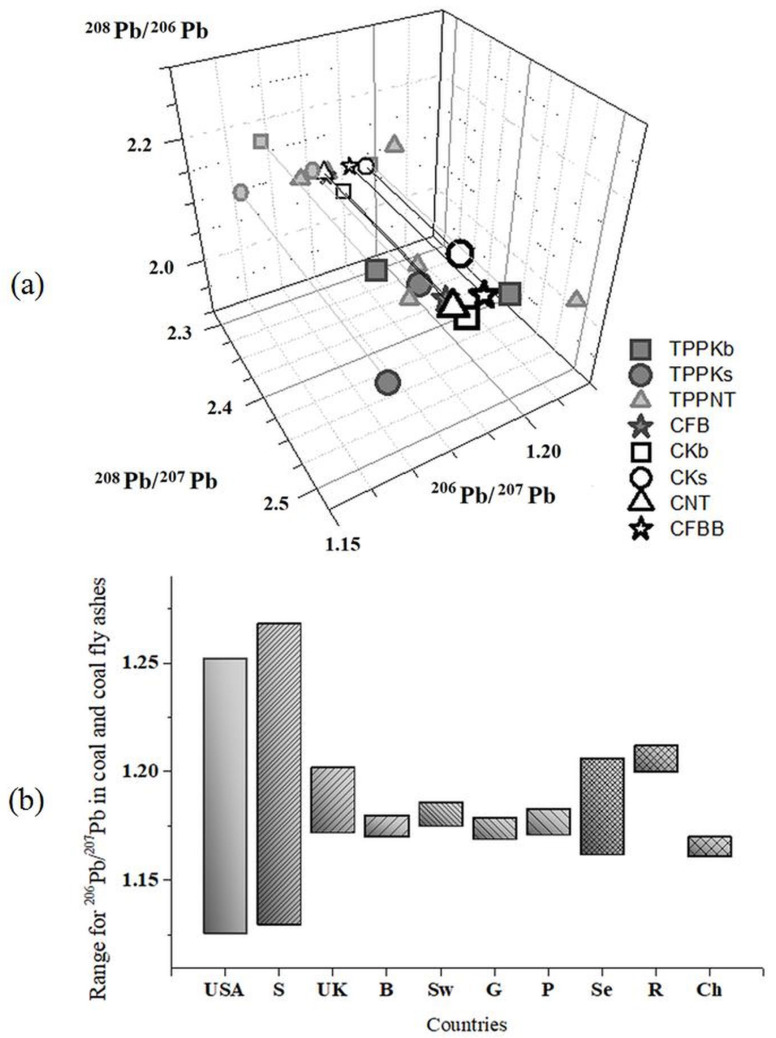
(**a**) Three-dimensional diagram of Pb isotope ratios in fly ashes (TPPKb, TPPKs, TPPNT, and CFB) and correspondent coals (CKb, CKs, CNT, and CFBB); (**b**) range for ^206^Pb/^207^Pb in different countries worldwide from west to east, i.e., USA: United States of America; S: Spain; UK: United Kingdom; B: Belgium; Sw: Switzerland; G: Germany; P: Poland; Se: Serbia; R: Russia; and Ch: China.

Coal combustion is a predominant source of lead pollution, and because of that lead isotopic composition in FAs and coals has a major influence on total Pb isotopic fingerprint [29]. Figure S1a demonstrates the ranges of lead isotope ratios among investigated fly ashes and coals for ^206^Pb/^207^Pb (from 1.162 to 1.206), ^208^Pb/^207^Pb (from 2.434 to 2.533), and ^208^Pb/^206^Pb (2.045 to 2.167). In addition, Figure S1b shows the values of ^208^Pb/^206^Pb and ^206^Pb/^207^Pb from available literature data [29,30,32,33]. The 3D diagram of all determined Pb isotope ratios for Serbian coals and fly ashes are shown in Figure 5a. Figure 5b compares ^206^Pb/^207^Pb literature data for coal combustion worldwide [29,33] with data obtained in this study. The mean value of ^206^Pb/^207^Pb ratio for Serbian coals and FAs is 1.186 ± 0.012, and it highly correlates with the United Kingdom (1.187), Switzerland (1.181), and the USA (1.189). Therefore, identifying the lead pollution fingerprint for Serbian coals and FAs contributes to lead pollution studies.

### 3.4. Trace Elements EFs

Table 2B also summarizes the EF values of trace elements, as well as their lower and upper limits along with the average value obtained from the EMEP inventory manual. Among investigated elements, EFs are considerably higher for As (FBB and TPP Kolubara A), Cr (FBB), Cu (TPP Kostolac B), Hg (TPPs Kostolac B and Nikola Tesla A), and Ni (TPP Kostolac B). Much higher EF values for some trace elements in all TPPs than their representative EFs [52,53] can be explained by the poorer quality of lignite utilized in all TPPs compared with coal provided in EMEP.

Despite having different powers, the EFs of Cr, Cu, Hg, and Pb in TPPs Kolubara A and Nikola Tesla A are comparable, at the same time TPP Kolubara A exhibits EFs higher from around 40% for As and Ni to 106% for Cd. Higher temperatures, better process optimization, and better air pollution management in TPPs, combined with lower feed fuel quality in FBB, result in much higher EFs of all trace elements (aside from Hg) for FBB than for TPPs (Table 2B).

Table S3 compares As, Cr, Hg, and Pb emission concentrations, ranging from 4.02 μg/m^3^ for Hg in FBB to 42.68 μg/m^3^ for Pb in TPP Kostolac B, to their respective emission limits for coal-fired units [60,61]. Arsenic emission concentrations from all combustion facilities exceed limit values compared with both standards (Table S3), while only Hg emission from FBB meets US EPA criteria.

### 3.5. Environmental Concerns of Investigated Trace Elements from FAs

#### 3.5.1. Crustal Enrichment Factor Normalized to Mn (*CEF_n/Mn_*)

**Figure 6:**
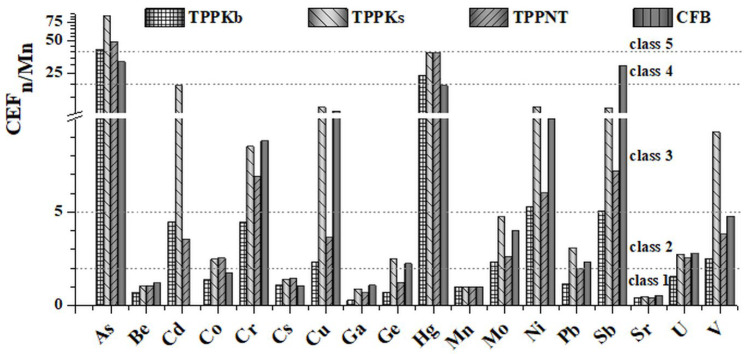
Crustal enrichment factor (*CEF_n/Mn_*) for fly ashes (TPPKb, TPPKs, TPPNT, and CFB).

*CEF_n/Mn_* values for all investigated elements were determined and presented in Figure 6. To comprehensively evaluate the pollution level of elements for examined FAs, five classes for CEF [62] were displayed in Table S4. Among investigated trace elements, As and Hg show the highest enrichment, which is more pronounced for all FAs from TPPs compared with CFB. CFB, on the other hand, has the greatest *CEF_n/Mn_* values among all FAs for Be, Cr, Ga, Sb, Sr, and U. No enrichment is observed for Be, Cs, Ga, and Sr.

#### 3.5.2. Risk Assessment Code

The toxic trace elements content in various matrices is typically expressed as total or water leaching concentrations. The content and leaching patterns of potentially toxic substances in coal combustion residues can provide valuable information for landfilling or be a limiting factor for application [63]. Table S5 compares the content of the most toxic elements in the investigated fly ashes with the European countries’ legislation. According to the Canadian Environmental Protection Act, six elements (As, Cd, Hg, Ni, Cr, and Pb) are defined as toxic substances [64]. As and Hg for all FAs from TPPs, as well as Pb for all FAs, are above limit values [65], while other potentially hazardous elements are below these limits. The trace elements from F1 fractions can be defined as non-hazardous [66], as shown in Table S5.

**Figure 7:**
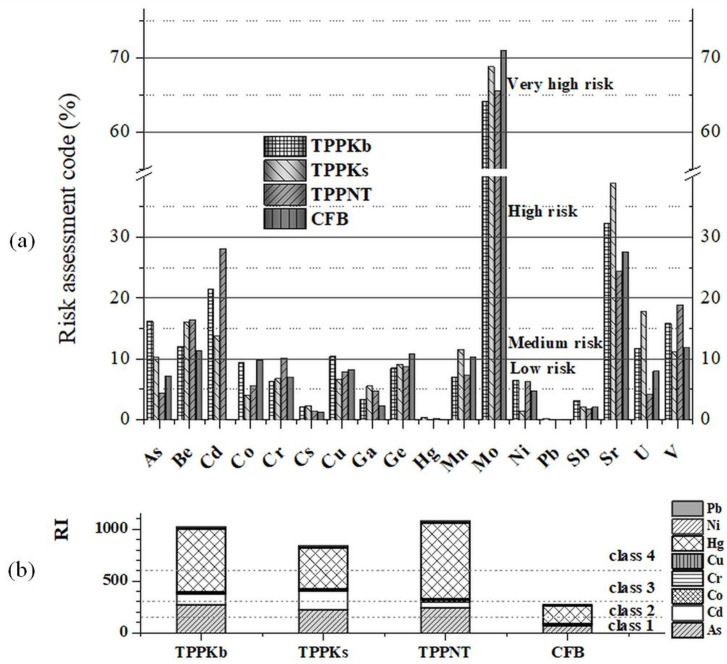
(**a**) Risk assessment code (RAC) and (**b**) risk index (RI) for fly ashes (TPPKb, TPPKs, TPPNT, and CFB).

RAC classification (Table S4) is important for estimating the potential leachability of elements from the sample matrix to the environment. The results for RAC (Figure 7a) suggest that Mo presents the highest risk. Sr displays medium risk of TPPNT and CFB, while values for TPPKb and TPPKs are above 30%. Be (up to 16.47%) and V (up to 18.93%) in all FAs, as well as Cd (up to 28.10%) for FAs from TPPs, pose a medium risk to the environment. Among investigated trace elements, only Co, Ge, and Mo have higher RAC values for CFB compared with the other FAs from TPPs. Although RAC values for some trace elements (Figure 7a) indicate a high environmental risk, some of them, such as Mo, are not considered environmentally relevant [14,65].

#### 3.5.3. Pollution Indices and Risk Indices

Table S6 summarizes PI values for all investigated trace elements along with their pollution levels [67]. PIs indicate very high pollution levels for As (from 11.25 in CFB to 42.17 in TPPKb) and Hg (from 6.71 in CFB to 29.35 in TPPNT), while Cr, Ni, and Sb show a high level of pollution (Table S6). Among investigated FAs, Be, Ga, and Sr do not pose any pollution (class 1), while only Sb shows the highest pollution in CFB compared with other FAs.

The potential ecological risk index (RI) categorizes different matrices based on the degree of contamination (Table S4 shows ecological risk limits). Figure 7b depicts RIs for the most toxic elements (As, Cd, Co, Cr, Cu, Hg, Ni, and Pb) in investigated fly ashes. Since the RI values for all FAs from TPPs are higher than 600, they represent a very high ecological risk, while CFB shows a moderate ecological risk [68,69].

### 3.6. PAHs Content in FAs

Individual and total PAH concentrations are presented in Table 3. Overall PAH contents are from 286.69 ng/g (TPPNT) to 33,378.53 ng/g (CFB), which is in accordance with the literature [19,28,70,71]. Figure S2 shows PAHs distribution by ring number (a) and the total and carcinogenic PAH contents of the investigated FAs (b). The four ring PAHs have the highest yield in CFB (68.29%) and TPPKb (66.07%), while the sum of two and three ring PAHs predominates in TPPKs (75.44%) and TPPNT (68.16%). Flu and Fla are the most abundant among examined PAHs (Table 3), as expected, since they can be commonly found in products of incomplete combustion of fossil fuels [70].

### 3.7. PAH Emission Factors

PAHs belong to the most hazardous and persistent organic compounds, particularly BbF, BkF, BaP, and IP, and since they can easily reach the atmosphere, estimation of their EFs is important [72]. Additionally, Table 2C shows PAHs emission for the combustion of lignite in TPPs and coal waste in FBB, as well as their EMEP emission limits [45].

PAHs portion in the finest ash particles emitted along with flue gases ranges from 39.96% (TPPs Kostolac B and Nikola Tesla A) to 94.63% (FBB). TPPs Kostolac B and Nikola Tesla A have BaP emission that exceeds the upper limit specified by EMEP, most likely due to lower lignite quality than coal listed in the EMEP (Table 2C). BbF, BkF, BaP, and IP emissions from TPP Kolubara A are within permissible limits. The estimated EF values for FBB (Table 2C) are elevated probably due to reduced combustion temperature, poor coal quality, and low cyclone efficiency.

### 3.8. Potential Environmental Effects of PAHs from FAs

To assess the carcinogenic potential of studied FAs, calculated overall BaP_eq_ and BaPE are shown in Table 3. The total BaP_eq_ for PAHs ranges from 5.21 ng/g (TPPNT) to 876.73 ng/g (CFB), while BaPE varies from 3.66 ng/g to 632.15 ng/g for TPPKs and CFB, respectively. The calculated BaP_eq_ and BaPE values are in accordance with the literature findings for different FAs [28,73]. Figure S3 shows BaP_eq_ ratios for each PAH expressed relative to BaP (set as 100%). BaA proportions are the highest and range from 50.25% (TPPNT) to 150.83% (CFB).

## 4. Conclusions

The environmental impact of byproducts generated during coal combustion in various TPPs (Kolubara A, Kostolac B, and Nikola Tesla A) and coal waste burning in an experimental semi-industrial fluidized bed boiler was investigated in this study. The aim was to determine the emission of gaseous pollutants (NOx, CO, and SO_2_) and total PM, estimate trace elements and PAHs emission factors, assess the environmental risk of FAs landfilling, and establish lead isotope ratios for Serbian coals and FAs.

The general conclusions are:

- Total PM emission factors exceed EMEP PM10 upper limits for all combustion facilities, while significantly higher EFs values are noticed for NOx and SO_2_ in TPP Kolubara A and CO in TPPs Nikola Tesla A and Kostolac B;
- Arsenic emissions from all combustion facilities exceed the limit values specified in relevant standards, whereas only Hg emissions from FBB fulfill the criterion established by the US EPA;
- FBB has much higher BbF, BkF, BaP, and IP EF values than TPPs due to the poor quality of coal waste and combustion conditions in FBB;
- Water-soluble fractions have the lowest trace element concentrations, ranging from 9.35 mg/kg (CFB) to 22.45 mg/kg (TPPKb), whereas the highest carcinogenic trace element contents are in the residual fractions, varying from 39.52% (TPPNT) to 42.92% (CFB);
- The CEF_n/Mn_ and PIs for As and Hg imply higher enrichment and pollution levels, however their low RAC values indicate that they will not easily leach into the environment;
- All TPP fly ashes have RI values that show a very high ecological risk, while CFB has a moderate ecological risk;
- CFB has the highest BaP_eq_ and BAPE values, suggesting a significant carcinogenic potential and thus high environmental risk;
- The determined lead isotope fingerprint for investigated coals and FAs is within ranges of other countries and can be particularly useful in the source apportionment of lead pollution.

This paper provides comprehensive systematic research of the overall environmental impact of different combustion facilities from the emission and fly ash landfilling perspective. To reduce the environmental risk of ash disposal, it is important to monitor harmful trace elements, and persistent organic pollutants such as PAHs, as well as to enhance environmental pollution control in the Serbian energy sector.

## Figures and Tables

**Table 1:** Flue gas flows for TPPs and FBB under working conditions (f_W_), under standard conditions and dry flue gas (f_st_), and under standard conditions for dried flue gas and oxygen content of 6% (f_R_).

TPP	Kolubara A	Kostolac B	Nikola Tesla A	FBB
f_W_ (m^3^/h)	509,642.3	3,260,671.3	2,429,662.3	820
f_st_ (Nm^3^/h)	254,751.3	1,596,911.0	1,201,327.3	434
f_R_ (Nm^3^/h)	187,123.3	1,535,411.3	850,350.0	315

**Table 2:** Comparison of EF values for NOx, CO, SO_2_, and PM (A), EF values for As, Cd, Cr, Cu, Hg, Ni, and Pb (B) and EF values for BaP, BbF, BkF, and IP (C) determined for the combustion of lignite and coal waste with literature data.

			A				B							C			
Details about boiler			EFs (g/GJ)				EFs (mg/GJ)							EFs (mg/GJ)			
		Pollutant	NOx	CO	SOx	PM *	As	Cd	Cr	Cu	Hg	Pb	Ni	BbF	BkF	BaP	IP
Small non- residential sources 50 kW–1 MW; coal fuels [52]		Value	160	2000	900	190	5	3	15	30	7	200	20	130	50	100	40
		lower	150	200	450	76	0.5	1	1	8	5	80	2	17	8	13	6
		upper	200	3000	1000	240	8	5	20	50	9	300	30	180	100	150	80
Fluidized bed boiler; 500 kW; coal waste (this paper)			9.8	3703	74.6	841.7	31.8	/	40.5	56.7	7.4	72.2	33.4	306.4	33.4	210.7	79.8
**Details about boiler**			**EFs (g/GJ)**				**EFs (mg/GJ)**							**EFs (mg/GJ)**			
		**Pollutant**	**NOx**	**CO**	**SOx**	PM *	**As**	**Cd**	**Cr**	**Cu**	**Hg**	**Pb**	**Ni**	**BbF**	**BkF**	**BaP**	**IP**
Medium size non-residential sources; 1–50 MW; coal fuels [52]		Value	180	200	900	76	4	1	15	10	9	100	10	17	9	13	6
		lower	150	150	450	60	0.5	0.5	1	8	5	80	2	10	8	10	5
		upper	200	3000	1000	240	5	3	20	30	10	200	20	180	100	150	80
Thermal power plant Kolubara A; 32 MW; lignite (this paper)			597.2	66.7	2675.0	1225.6	11.7	0.4	19.2	7.9	12.7	12.9	27.7	0.4	0.1	0.1	0.1
**Details about boiler**			**EFs (g/GJ)**				**EFs (mg/GJ)**							**EFs (µg/GJ)**			
		**Pollutant**	**NOx**	**CO**	**SOx**	PM *	**As**	**Cd**	**Cr**	**Cu**	**Hg**	**Pb**	**Ni**	**BbF**	**BkF**	**BaP**	**IP**
Public power combustion plants (≥300 MW or 50–300 MW); brown coal/lignite [53]		Value	247	8.7	1680	7.9	14.3	1.8	9.1	1.0	2.9	15	9.7	37	29	1.3	2.1
		lower	143	6.7	330	1	10.3	1.3	6.6	0.2	2.1	10.6	7.1	3.7	2.9	0.3	0.4
		upper	571	60.5	5000	79	24.1	3	15.3	5	4.9	24.7	16.5	370	290	6.5	10.5
Thermal power plant Kostolac B; 350 MW; lignite (this paper)			233.3	305.6	7436.1	105.6	12.6	0.8	18.8	20.7	10.6	17.8	32.9	22.5	68.4	15.1	4.1
Thermal power plant Nikola Tesla A; 210 MW; lignite (this paper)			66.7	533.3	2311.1	300.0	8.5	0.2	18.2	7.5	12.6	13.4	19.3	8.0	7.6	17.5	1.0

* PM stands for PM10 [52,53] or for total PM (this paper).

**Table 3:** PAHs content in fly ashes (TPPKb, TPPKs, TPPNT, and CFB).

PAH		Abbreviation	Fly Ashes (ng/g)			
			TPPKb	TPPKs	TPPNT	CFB
Naphthalene		Nap	23.54	10.88	7.33	21.80
1-methyl naphthalene		1mNap	55.56	3.90	6.60	274.10
2-methyl naphthalene		2mNap	10.78	0.00	1.14	555.77
Acenaphthylene		Acy	2.77	49.36	38.05	745.23
Acenaphthene		Ace	6.56	8.29	3.25	104.70
Fluorene		Flu	150.06	144.76	92.01	1576.70
Phenanthrene		Phe	385.87	129.26	44.90	4993.90
Anthracene		Ant	17.20	1.87	2.14	1159.98
Fluoranthene		Fla	795.89	55.31	50.24	9509.68
Pyrene		Pyr	410.35	13.01	14.17	6999.46
Benz[a]anthracene		BaA	84.37	20.39	15.32	3838.38
Chrysene		Chry	77.25	8.89	4.09	2445.81
Benzo[b]fluoranthene		BbF	25.05	2.50	1.39	491.95
Benzo[k]fluoranthene		BkF	4.32	7.61	1.33	53.56
Benzo[a]pyrene		BaP	5.59	1.68	3.05	338.25
Dibenz[a,h]anthracene		DahA	0.04	0.02	0.00	25.29
Benzo[g,h,i]perylene		BghiP	10.66	3.54	1.49	115.92
Indeno[1,2,3-cd]pyrene		IP	4.51	0.46	0.17	128.05
	Total PAHs		2070.38	461.72	286.69	33,378.53
	Sum of 10 PAHs *		1434.26	242.38	131.46	23,097.28
	Total BaP_eq_		20.35	5.36	5.21	876.73
	Total BAPE		13.10	3.66	4.17	632.15

* The sum of ten hazardous PAHs (Ant, BaA, BbF, BkF, BaP, Chry, Phe, Fla, IP, and BghiP) that have soil guideline limits in Serbia.

## Data Availability

The data presented in this study are available on request from the corresponding author.

## Supplementary Materials

The following supporting information can be downloaded at https://www.mdpi.com/article/10.3390/toxics11040396/s1:, Figure S1: (a) Lead isotope ratios (^206^Pb/^207^Pb and ^208^Pb/^207^Pb *vs.* ^208^Pb/^206^Pb) for all investigated samples from Serbia (8 fly ashes and 4 coals); (b) Lead isotope ratios (^206^Pb/^207^Pb *vs.* ^208^Pb/^206^Pb) depending on originating country; Figure S2: (a) PAHs proportions by their ring number (R2-R6); (b) The total and carcinogenic PAHs content for fly ashes (TPPKb, TPPKs, TPPNT and CFB); Figure S3: The individual BaP_eq_ ratios relative to BaP (%); Table S1. Locations of combustion facilities and lignite mining basins; Table S2: Limit values of NOx, CO, SO_2_ and total PM concentrations (mg/Nm^3^) in flue gases of combustion facilities with different capacities; Table S3: Flue gas emissions of trace elements (μg/m^3^); Table S4: Adjusted indices of element enrichment degree, environmental risk and ecological risk; Table S5: Comparative view of total heavy metal concentrations and their water leachates with literature data; Table S6: Pollution indices (PIs) of investigated trace elements for all FAs.

## Abbreviations

BA	bottom ash
BaP	benzo[a]pyrene
BaPE	benzo[a]pyrene-based equivalent carcinogenic power
BaP_eq_	benzo[a]pyrene-based toxic equivalent factor
BbF	benzo[b]fluoranthene
BkF	benzo[k]fluoranthene
C	carcinogenic elements
CEF	crustal enrichment factor
CFB	fly ash from cyclone of fluidized bed boiler
CFBB	coal waste from fluidized bed boiler
CKb	coal from TPP Kolubara A
CKs	coal from TPP Kostolac B
CNT	coal from TPP Nikola Tesla A
EF	emission factor
EMEP	European monitoring and evaluation programme
F1	water-soluble fraction
F2	exchangeable fraction
F3	carbonate bound fraction
F4	metal oxide bound fraction
F5	organic bound fraction
F6	residual fraction
FA	fly ash
FBB	fluidized bed boiler
ICP-MS	inductively coupled plasma mass spectrometry
IP	indeno[1,2,3-cd]pyrene
NC	non-carcinogenic elements
PAHs	polycyclic aromatic hydrocarbons
PI	pollution index
PM	particulate matter
RAC	risk assessment code
RI	risk index
TPP	thermal power plant
TPPKb	fly ash from TPP Kolubara A
TPPKs	fly ash from TPP Kostolac B
TPPNT	fly ash from TPP Nikola Tesla A
