# Supplementary material for: Risk Evaluation of Pollutants Emission from Coal and Coal Waste Combustion Plants and Environmental Impact of Fly Ash Landfilling
Source: Toxics. 2023 Apr 21;11(4):396. doi: 10.3390/toxics11040396 (PMC10144006; doi:10.3390/toxics11040396)
Supplement: Supplementary file 1 [file toxics-11-00396-s001.zip › toxics-2312736-supplementary final.pdf]

toxics MDPI

Article

Risk Evaluation of Pollutants Emission from Coal and Coal Waste Combustion Plants and Environmental Impact of Fly Ash Landfilling

Jovana Z. Buha Marković [1,*], Ana D. Marinković [1], Jasmina Z. Savić [1], Milica R. Mladenović [1], Milić D. Erić [1], Zoran J. Marković [1] and Mirjana Đ. Ristić [2]

[1] Vinča Institute of Nuclear Sciences—National Institute of the Republic of Serbia, University of Belgrade, Mike Petrovića Alasa 12–14, 11351 Belgrade, Serbia
[2] Faculty of Technology and Metallurgy, University of Belgrade, Karnegijeva 4, 11120 Belgrade, Serbia

Supplementary material

S. MATERIALS AND METHODS

S1. Ash sample collection and storage

Fly ash sampling locations at four combustion facilities, as well as the locations of lignite mining basins are shown in Table S1.

Table S1. Locations of combustion facilities and lignite mining basins.

Combustion Facility	Location	Installed Capacity (MW)	Lignite Source
TPP Kolubara A	44° 28′ 51″ N, 20° 17′ 36″ E	32	KB
TPP Kostolac B	44° 43′ 41″ N, 21° 12′ 50″ E	350	DB
TPP Nikola Tesla A	44° 40′ 20″ N, 20° 9′ 22″ E	210	KB
Fluidized bed boiler	44° 45′ 20″ N, 20° 36′ 10″ E	0.5	KB (TPP Kolubara)
**Lignite mining basin**	**Location**		
Kolubara basin (KB)	44° 30′ 38″ N, 20° 7′ 32″ E		
Drmno basin (DB)	44° 42′ 27″ N, 21° 14′ 51″ E		

S2. Sequential extraction procedure

The following fractions of trace elements were extracted in a sequential extraction procedure:

- **Water soluble fraction—F1**: 5 g of ash sample was taken in polyethylene bottles and shaken with 50 mL of pure Millipore water for 3 h at room temperature. After that they were filtered using Whatman filter paper 42 and the filtrate was kept for further analysis.
- **Exchangeable fraction—F2**: the residue from the previous step was treated with 8 mL of 1 M $MgCl_2$, shaken for 1 h at room temperature and filtered on Whatman filter paper 42. The filtrate was kept for further analysis.
- **Carbonate bound fraction—F3**: the residue from the previous step was mixed on an orbital shaker with 50 mL of 1 M sodium acetate at pH 5 for 1 h at room temperature and filtered on Whatman filter paper 42. The filtrate was kept for further analysis.
- **Metal oxide bound fraction—F4**: the residue obtained from the previous step was treated with 50 mL of 0.04 M hydroxylamine hydrochloride in 25% acetic acid and digested for 5 h on a hot plate at 96°C. After digestion, the

solution was filtered on Whatman filter paper 42. The filtrate was kept for further analysis.

- **Organic bound fraction—F5**: the residue from the previous step was treated with 10 mL of 30% H_2O_2 and 10 mL 0.02 M HNO_3 and after that was heated at 85°C for 3 h, covered with a watch glass and digested for 1 h. After the solution got vaporized the residue was cooled. 20 mL of 3.2 M CH_3COONH_4 in 20% HNO_3 was added and shaken continuously for 30 min. The solution was filtered on Whatman filter paper 42. The filtrate was kept for further analysis.
- **Residual fraction—F6**: 0.2 g of residue was subjected to microwave digestion with 8 mL of aqua regia, up to 220°C with a heating rate of 5°C/min, and then held for 15 min at 220°C. The filtrate was diluted by distilled water to 50 mL and analyzed.

S3. ICP-MS Analysis

The instrument optimization was done with a tuning solution containing 1 μg/L Li, Mg, Co, Y, Ce and Tl (Agilent). The limits of detection of measured elements were (μg/L): As 0.015, Be 0.046, Cd 0.002, Co 0.001, Cr 0.015, Cs 0.01, Cu 0.005, Ga 0.007, Ge 0.006, Hg 0.01, Mn 0.098, Mo 0.07, Ni 0.022, Pb 0.005, Sb 0.01, Sr 0.163, U 0.005, V 0.001. Quality control was performed using the certified reference material SLRS-5. Accuracy was in the range of 88–112%.

Figures:

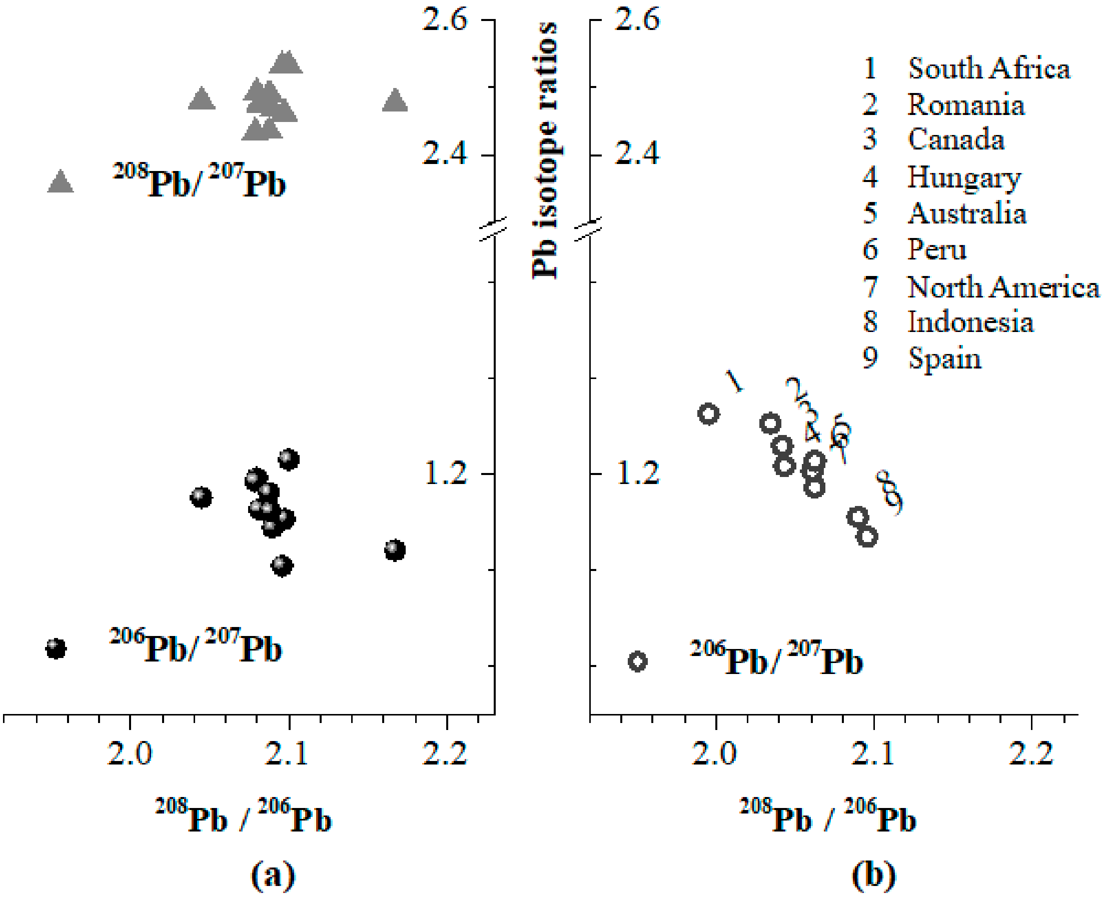

Figure S1. (**a**) Lead isotope ratios (^{206}Pb / ^{207}Pb and ^{208}Pb / ^{207}Pb vs. ^{208}Pb / ^{206}Pb) for all investigated samples from Serbia (8 fly ashes and 4 coals); (**b**) Lead isotope ratios (^{206}Pb / ^{207}Pb vs. ^{208}Pb / ^{206}Pb) depending on the originating country.

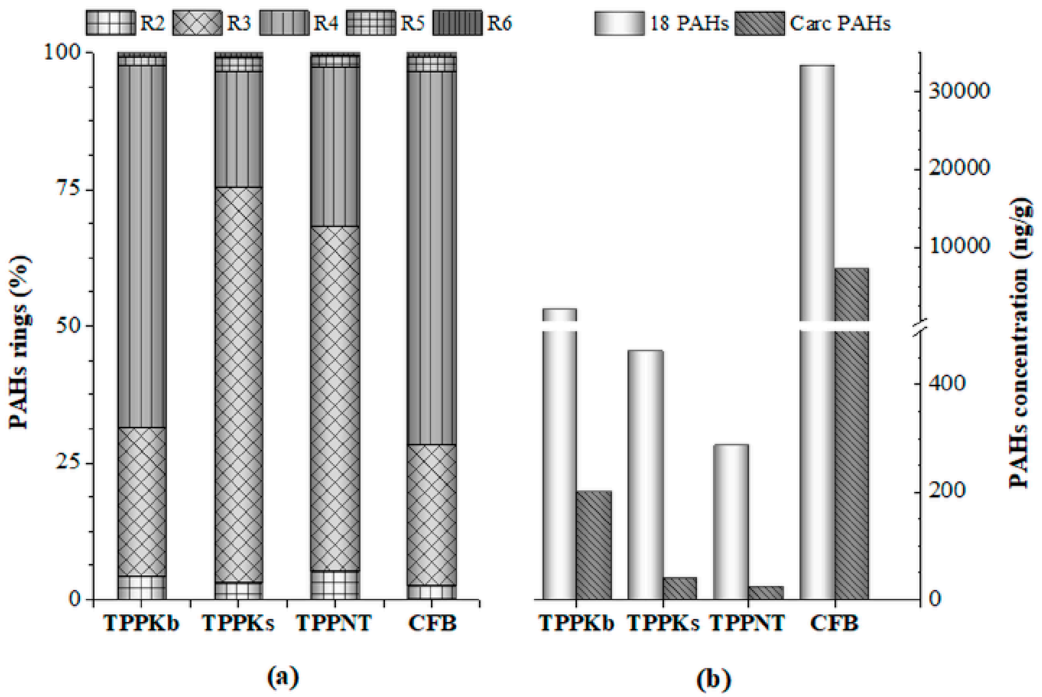

Figure S2. (**a**) PAHs proportions by their ring number (R2-R6); (**b**) The total and carcinogenic PAHs content for fly ashes (TPPKb, TPPKs, TPPNT and CFB).

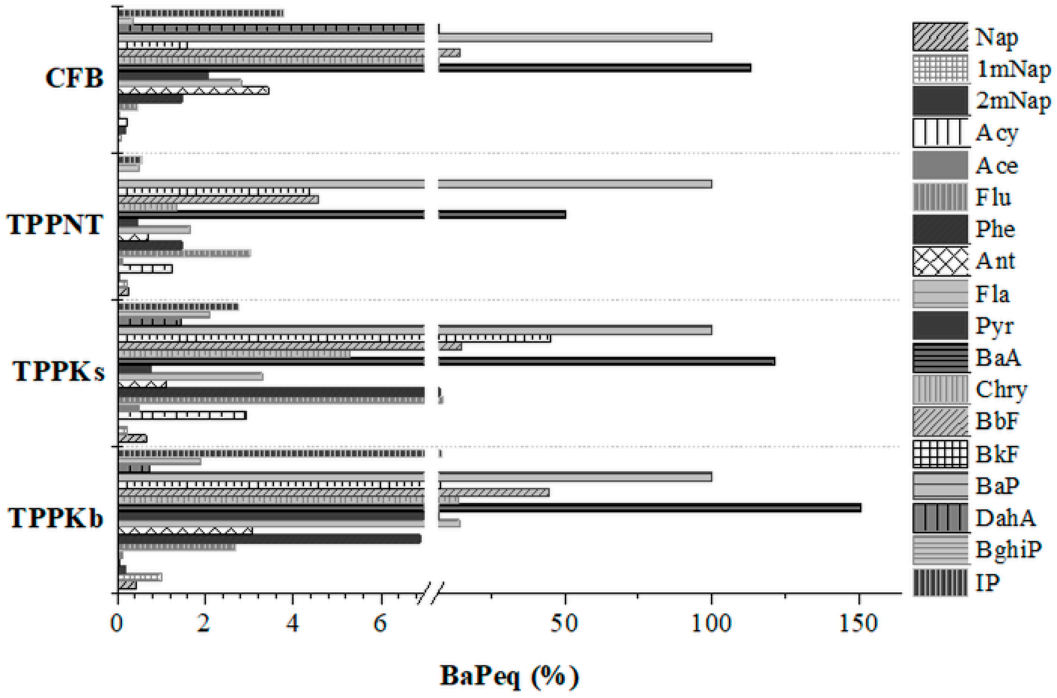

Figure S3. The individual BaP_{eq} ratios relative to BaP (%).

Tables:

Table S2. Limit values of NOx, CO, SO_2 and total PM concentrations (mg/Nm^3) in flue gases of combustion facilities with different capacities.

Combustion Facility	Pollutant				Source
	NOx	CO	SO_2	total PM	
TPP Nikola Tesla A	500	250	400	50	[2]
TPP Kostolac B	500	250	400	50	[2]
TPP Kolubara A	600	250	1880	1010	[2]
FBB*	/	2000	/	150	[2]

* Limit values for low capacity combustion facilities are expressed for 8% O_2

Table S3. Flue gas emissions of trace elements (μg/m^3).

Combustion Facility	As	Cr	Hg	Pb	Source
TPP Nikola Tesla A	11.23	7.26	10.23	27.30	This paper
TPP Kostolac B	19.44	8.83	10.17	42.68	This paper
TPP Kolubara A	9.20	4.57	6.17	15.66	This paper
FBB	5.80	7.37	4.02	26.27	This paper
Emission standard	2.00	2.54	5.08	3.81	[3]
	2.54	900	30	80	[4]

Table S4. Adjusted indices of element enrichment degree, environmental risk and ecological risk.

Class	CEF	Enrichment degree [5]	Class	RAC (%)	Environmental Risk [6]	Class	RI	Ecological Risk [7]
**1**	< 2	no enrichment	**1**	< 1	safe for the environment	**1**	< 150	low
**2**	2-5	moderate	**2**	1-10	low risk	**2**	150-300	moderate
**3**	5-20	significant	**3**	11-30	medium risk	**3**	300-600	considerable
**4**	20-40	high	**4**	31-50	high risk	**4**	> 600	very high
**5**	> 40	very high	**5**	> 50	very high risk			

Table S5. Comparative view of total heavy metal concentrations and their water leachates with literature data

Samples	Total Concentration (mg/kg)							Water Leachates (mg/kg)						
	As	Cd	Cr	Cu	Hg	Ni	Pb	As	Cd	Cr	Cu	Hg	Ni	Pb
**TPPKb**	63.0	0.44	158	62	1.77	107	23	2.83	0.004	1.5	0.70	n.d.	n.d.	n.d.
**TPPKs**	52.8	0.76	121	122	1.10	99	25	0.61	n.d.	1.2	0.10	n.d.	n.d.	n.d.
**TPPNT**	56.3	0.26	183	70	2.00	91	29	0.36	n.d.	1.7	0.13	n.d.	n.d.	n.d.
**CFB**	16.9	n.d.	108	98	0.50	70	16	0.38	n.d.	1.4	n.d.	n.d.	n.d.	n.d.
**Limit values* (mg/kg)**	30	17.5	300	700	1	150	15	n.d. – not detected						
**Water leachates** (mg/kg)**							**NH**	2	1	10	50	10	10	
							**H**	25	5	70	100	40	50	

* heavy metal maximal permissible concentrations in ashes used in agriculture and as a forest fertilizer—Finland legislation [8]

** limit values for water leachates of ashes (NH-non-hazardous, H-hazardous)—European legislation [9]

Table S6. Pollution indices (PIs) of investigated trace elements for all FAs.

element	PI			
	TPPKb	TPPKs	TPPNT	CFB
As	42.17	35.19	37.51	11.25
Be	0.70	0.42	0.79	0.42
Cd	4.53	7.77	2.69	n.d.
Co	1.40	1.00	1.92	0.61
Cr	4.51	3.45	5.23	3.09
Cs	1.10	0.57	1.10	0.37
Cu	2.37	4.86	2.75	3.90
Ga	0.30	0.36	0.52	0.38
Ge	0.71	1.02	0.92	0.79
Hg	24.30	15.81	29.35	6.71
Mn	1.01	0.41	0.76	0.35
Mo	2.36	1.93	1.98	1.41
Ni	5.36	4.94	4.54	3.50
Pb	1.16	1.25	1.47	0.82
Sb	5.12	4.80	5.45	10.33
Sr	0.43	0.20	0.33	0.19
U	1.57	1.11	1.91	0.98
V	2.53	3.76	2.89	1.68

class	ranges	pollution level	class	ranges	pollution level
1	PI < 1	none	4	3 ≤ PI < 5	high
2	1 ≤ PI < 2	low	5	PI ≥ 5	very high
3	2 ≤ PI < 3	moderate			

References

1. Buha-Marković, J.Z.; Marinković, A.D.; Nemoda, S.Đ.; Savić, J.Z. Distribution of PAHs in coal ashes from the thermal power plant and fluidized bed combustion system; estimation of environmental risk of ash disposal. *Environmental Pollution* **2020**, *266*, 115282. https://doi.org/10.1016/j.envpol.2020.115282.
2. Government of the Republic of Serbia. Regulation on Emission Limit Values for Gaseous Pollutants from the Combustion Plants; 110-13947/2015-1; Government of the Republic of Serbia: Belgrade, The Republic of Serbia, **2016**.
3. U.S. EPA, *National emission standards for hazardous air pollutants: coal- and oil-fired electric utility steam generating units; Subcategory of certain existing electric utility steam generating units firing eastern bituminous coal refuse for emissions of acid gas hazardous air pollutants*; US EPA: Durham, N.C., **2020**.
4. Ministry of Environmental Protection of China (MEPC). Emission standard of air pollutants for coal-fired power plants. Ministry of Environmental Protection of China (MEPC): Bejing, China, **2011**.
5. Zhu, H.-n.; Yuan, X.-z.; Zeng, G.-m.; Jiang, M.; Liang, J.; Zhang, C.; Yin, J.; Huang, H.-j.; Liu, Z.-f.; Jiang, H.-w. Ecological risk assessment of heavy metals in sediments of Xiawan Port based on modified potential ecological risk index. *Transactions of Nonferrous Metals Society of China* **2012**, *22*, 1470–1477. https://doi.org/10.1016/S1003-6326(11)61343-5.
6. Zhou, Y.; Ning, X.-a.; Liao, X.; Lin, M.; Liu, J.; Wang, J. Characterization and environmental risk assessment of heavy metals found in fly ashes from waste filter bags obtained from a Chinese steel plant. *Ecotoxicology and Environmental Safety* **2013**, *95*, 130–136. https://doi.org/10.1016/j.ecoenv.2013.05.026.
7. Hakanson, L. An ecological risk index for aquatic pollution control. A sedimentological approach. *Water Research* **1980**, *14*, 975–1001.https://doi.org/10.1016/0043-1354(80)90143-8.
8. Dahl, O.; Nurmesniemi, H.; Pöykiö, R.; Watkins, G. Heavy metal concentrations in bottom ash and fly ash fractions from a large-sized (246MW) fluidized bed boiler with respect to their Finnish forest fertilizer limit values. *Fuel Processing Technology* **2010**, *91*, 1634–1639.https://doi.org/10.1016/j.fuproc.2010.06.012.
9. ECD. Council decision of 19 December 2002 establishing criteria and procedures for the acceptance of waste at landfills pursuant to Article 16 of and Annex II to Directive 1999/31/EC. *Official Journal of the European communities* **2002**, *16*, 27–49.
